# Potential Bacterial Biomarkers Associated with *Penaeus stylirostris* Shrimp Larvae to Infer Holobiont Health and Dysbiosis Across Larvae Stages

**DOI:** 10.3390/microorganisms13112452

**Published:** 2025-10-25

**Authors:** Nolwenn Callac, Carolane Giraud, Valérie Perez, Dominique Ansquer, Jean-Sébastien Lam, Viviane Boulo, Dominique Pham, Nelly Wabete

**Affiliations:** 1 Ifremer, IRD, Université de la Nouvelle-Calédonie, Université de La Réunion, UMR 9220 ENTROPIE, Nouméa 98800, New Caledonia; carolanegiraud95@gmail.com (C.G.); valerie.perez@ifremer.fr (V.P.); dominique.ansquer@ifremer.fr (D.A.); jean.sebastien.lam@ifremer.fr (J.-S.L.); viviane.boulo@ifremer.fr (V.B.); dominique.pham@ifremer.fr (D.P.); nelly.wabete@ifremer.fr (N.W.); 2 Ifremer, MASAE Microbiologie Aliment Santé Environnement, 44000 Nantes, France; 3 Interactions Hôtes Pathogènes Environnements (IHPE), Université de Montpellier, CNRS, Ifremer, Université de Perpignan Via Domitia, 34090 Montpellier, France

**Keywords:** microbiome, pathobiome, active microbiota, shrimp larvae rearing, biosurveillance

## Abstract

Microbiota play a pivotal role in holobionts, influencing nutrient intake, growth, and overall health. In this context, microbial dysbiosis of *Penaeus stylirostris* larvae seem to be associated with huge larval mortalities in hatcheries in New Caledonia. To understand larval dysbiosis establishment, our purpose was to identify bacterial biomarkers, as bioindicators, related to a given larval stage and health condition. To this end, larvae were sampled daily to access their active microbiota through sequencing of the V4 region of the 16S rRNA molecule, while their stage and their health were also observed. We identified three biomarkers strongly related to healthy zoea, and some may act as probiotics or play key roles in larval ontogeny and nutrition. We also found six biomarkers linked to unhealthy zoea and eight related to healthy mysis. Biomarkers were mostly related to diseased shrimps (*Lewinella*) or healthy shrimps (*Cognitishimia*, *Thalassolituus*) or were known to prey on cells (P30B-42), suggesting that the larvae might be battling against detrimental conditions. No biomarker related to unhealthy mysis was identified. Finally, our data showed that bacterial bioindicators could be used as an effective biosurveillance proxy in hatcheries, to monitor larval development, and as an early warning tool to predict rearing outcomes.

## 1. Introduction

Many species of eukaryotic macroorganisms host symbiotic microorganisms that colonize and inhabit various tissues or organs [1,2,3]. Depending on the compartments in which they live, symbionts are involved in various processes and influence physiological parameters such as digestion, nutrient intakes, growth, fitness, and the immune system response [4,5,6,7,8]. They therefore comprise a key component of host health. Complex interactions occur, and microbiota can colonize their host through various mechanisms: horizontal and vertical transmissions, co-evolution, and host modulation via biotic and abiotic interactions within the host and with the environment [1,2,9]. Together, the host and its symbionts form a holobiont [2,10]. In the case of microbiota imbalance, a change in microbial diversity, distribution, or metabolic activities can occur. Therefore, in the event of dysbiosis, their relationship can shift from mutualistic to detrimental. Therefore, dysbiosis of the holobiont can lead the healthy microbiota into a pathobiont [11,12,13,14]. A healthy microbiota is often described as stable and resilient and as symbiotic with the host [15]. It can also be defined by its high taxonomic diversity and microbial gene richness, as well as by a stable core microbiota [16]. Thus, disruption of the healthy microbiota, through the loss of beneficial organisms and/or the overgrowth of r-strategist microorganisms or detrimental pathogens, can induce diseases [11,12,13]. Various stressors can promote dysbiosis of the holobiont, such as environmental stress (sub- or infra-optimal temperature, for example) [17,18]. Regarding aquacultured species, rearing conditions (semi or intensive modes, feeds, etc.) can facilitate the fast spreading of diseases, potentially leading to disease outbreaks or polymicrobial infections [19,20,21]. Therefore, dysbiosis—particularly if it leads to diseases or polymicrobial infections—can prove a significant threat to rearings. Consequently, care must be taken in distinguishing beneficial from detrimental microbiota in aquacultured species. Current efforts in marine aquacultured shrimps aim to identify microbial health indicators or biomarkers as proxies of shrimp health or disease. However, most of the research has so far focused on *Penaeus vannamei* adults or larvae [7,22,23,24,25,26,27,28,29], and less on *P. stylirostris* [30,31,32]. So far, biomarkers as health indicators of *P. stylirostris* larvae have either been detected in the rearing water experiencing larval mortalities [31,33] or have been associated with healthy larvae [30,32].

In New Caledonia, shrimp production is based on the rearing of one species, the Pacific blue shrimp *Penaeus stylirostris*. The production of *P. stylirostris* is important for the territory’s socioeconomy as the industry is the first agri-food exporter, and it employs more than 500 people [32]. However, shrimp production has been facing issues in supplying farms with post-larvae over the past two decades (since 2005) due to a dramatic drop in larval production in hatcheries [34]. The main consequences are the disruption of both shrimp farming and the shrimp industry and, consequently, of the Neo-Caledonian economy. To date, despite numerous bacterial analyses, which have been performed by the Neo-Caledonian Network of Shrimp Epidemiological Vigilance (REC-DAVAR) at each episode of larval mortality, no cases of larval septicemia, viral infection, or parasitic infection have been detected—at least among the reportable diseases [21,35]. These results refute microbial infection, therefore arguing for either new pathologic agents or multifactorial causes [33]. As it might be difficult to look for specific etiologic agents, seeking bacterial biomarkers as larval health bioindicators might be helpful in monitoring the larval rearing in hatcheries and developing bio-surveillance tools for predicting rearing fate. This is quite important as there are only three active hatcheries in New Caledonia, which need to sell post-larvae to 17 farms (at the time of writing); therefore, measures must be taken to maintain shrimp production and its economic contribution to the territory. Consequently, our working hypothesis was that the larval microbiota could be influenced by the health status of the larvae, along with larval ontogeny linked to their developmental stage. Indeed, the microbiota of unhealthy larvae are expected to differ from those of healthy larvae, as observed for *Penaeus vannamei* [22]. Also, it has been shown that many lineages of healthy *P. stylirostris* are stage-specific, with some of these taxa being detected in natural seawater. It has also been shown that a common core microbiota exists for all stages—from eggs to mysis [30]. This indicates that larvae can modulate their microbiota and acquire bacterial lineages through vertical and horizontal transmissions [30]. To investigate the onset of dysbiosis, our aim was to identify the bacterial larval health bioindicators that are associated with specific larval stages and health conditions. To achieve this, we monitored rearings with different survival rates. All rearings were conducted using the same egg pool to avoid broodstock-related variability, but under different water treatments. Our results highlight that larva–microbiota interactions are influenced by both ontogeny and health status. Biomarkers of the healthy zoea appeared to be potentially beneficial for the larvae and could be involved in ontogeny and nutrition, or function as potential probiotics. Biomarkers of the unhealthy zoea and healthy mysis seemed to be gathered into three groups: those related to diseased shrimps; those related to healthy shrimps; and those that are context-specific (known to be part of the microbiota of healthy or unhealthy animals). Notably, no strong biomarkers related to unhealthy mysis were in evidence. Overall, our data suggest that bacterial biomarkers could be used as bioindicators for biosurveillance and as an early warning tool for monitoring larval health and predicting rearing outcomes in hatcheries.

## 2. Materials and Methods

### 2.1. Rearing Experiment

Shrimp eggs and larvae were collected during experimental rearings that took place in February 2019 in an experimental hatchery in Boulouparis (Station Aquacole de Saint Vincent), New Caledonia. Larvae were obtained after artificial inseminations of the same batch of mature *P. stylirostris* breeders as described by Pham et al. (2012) [36] to avoid the influence of breeders on survival. After spawning and hatching, nauplii from the same reproduction day were separated from egg debris and unhatched eggs, rinsed, and pooled into the same batch prior to their transfer into the rearing tanks on day 0 (D0). Rearings were set up according to different water treatments, namely, different seawater filtration pore sizes and addition, or not (as detailed in Callac et al., 2024 [30] and Table S1), of antibiotic (Erythromycin). In all rearing tanks, 5 g·m^−3^ of ethylenediaminetetraacetic acid (EDTA) was added on D0. Feeding frequency and food were dependent of the larval stage, with microparticles (size 5 to 50 µm) distributed five times per day and frozen *Tetraselmis* sp. once a day for zoea 1 and 2 m with flakes (size 50 to 100 µm) twice a day and living *Artemia* sp. for zoea3 to post-larvae. Also, no water exchange was applied during the larval phase.

Larval stage, health, and survival rates were monitored twice a day via direct observation and counting under binocular magnifying glass [33]. Larval activity was considered normal if larvae were active, with normal feeding behavior, evidence of digestive content in their gut, and the absence of visual signs of diseases [22,32]. Therefore, rearings were considered healthy if larvae were active and if the survival rates were above or equal to our reference [31,33]. The reference was determined for a given day using the data of successful rearing for a period of 10 years (2008–2018), for which no apparent and undetermined massive larval mortality was noticed. To be considered a healthy rearing, the survival rate must be superior or equal to the daily reference, as follows: D0 = 100%; D1 between 95 and 100%; D2 ≥ 95%; D3 ≥ 90%; D4 ≥ 85%; D5 ≥ 80%; D6 ≥ 77%; D7 ≥ 75%; D8 ≥ 73%; and D9 ≥ 70%.

### 2.2. Sample Collection, Total RNA Extractions, Reverse Transcription, and Sequencing

Using sterilized pliers, about 100 eggs were sampled after spawning and stored in a 2 mL sterile microtube, and 100 nauplii were collected using a 120 µm pore size net and a sterilized spatula and stored in 2 mL microtubes. Then, daily, before the first feeding, around 100 larvae were sampled using the same protocol as for the nauplii. One sample corresponds to the collection of 100 individuals per tank and per day, irrespective of the larval health. Then, each sample was processed separately. All samples were stored at −80 °C until RNA extractions. RNA from all the samples were extracted using the RNeasy minikit (Qiagen, Hilden, Germany) following the manufacturer’s instructions. RNA quantity and purity were controlled by NanoDrop measurements (One C Spectrophotometer UV-VIS, Fisher™, Waltham, MA, USA). Complementary DNA (cDNA) was obtained after reverse-transcription of the total RNA using the reverse transcriptase M-MLV (Promega, Madison, WI) and random hexamers, as described previously [30,31,32,33,37,38]. The cDNAs were shipped to MrDNA to produce amplicon of the V4 hypervariable region of the 16S rRNA gene using the 515f-806R primers [39,40] prior to Illumina HiSeq sequencing using a 2 × 150 paired-end run.

### 2.3. Bioinformatics and Downstream Analysis

Raw data were processed using the DADA2 package [41] in Rstudio software (using R version 4.4.1 in RStudio version 2024.04.1 + 748.). Sequences with a quality score greater than 30 were kept then filtered according to several parameters, as described in Callac et al. (2023) [31], maximum expected error (maxEE) was set at 2, maximum N (maxN) was set at 0, and chimeras were removed via the consensus method. Taxonomy was assigned using the Silva 138 SSU Ref NR99 database [42]. Prior to microbial diversity analysis, reads with no affiliation or affiliated with the *Eukaryota*, *Mitochondria*, or *Chloroplasts* were removed from the ASV table. The biggest library was 629,505 reads (sample NTA0L_D9C: larvae collected on D9 from tank C reared with water NT and erythromycin added for the first on D0), and the smallest was 21,246 reads (sample TA3L_D7C: larvae collected on D7 from the tank C reared with water T and antibiotic added on D3 for the first time); therefore, the data was normalized using the CPM (Count Per Million) method. This method, generally used to normalize gene expression data, was used in our study as we worked with RNAs that were reverse-transcribed into cDNA.

### 2.4. Downstream Data Analysis

All analyses were conducted using Rstudio (version 2024.04.1 + 748.) implemented in R (version 4.4.1) using the normalized ASV table. Normalization was performed using Phyloseq package (version 1.48.0) [43]. A heatmap to highlight the 40 most abundant bacterial genera was generated with microeco [44]. Three methods were used to identify biomarkers at the genus level that were specific to a given health condition, thus highlighting the genera involved in larval health and in larval dysbiosis. ANCOM-BC (Analysis of Compositions of Microbiomes with Bias Correction) was conducted based on count data using the whole ASV table and the ANCOMBC package (version 2.6.0) [45]. LEfSe, or Linear Discriminant Analyses (LDAs) of effect size [46], were performed, with a threshold set at 4 using the MicrobiomeMarker package (version1.10.0) [47]. Random forests, a machine learning method based on decision trees to classify microbial communities [48], were produced using the randomForest package (version 4.71.2) [49]. We set the main genera (predictors) at 10, allowing us to classify larvae (zoea or mysis) into healthy or unhealthy categories. Then, using the microeco package (version 1.14.0) [44], a correlogram based on Spearman correlation was built between the genera identified with ANCOM-BC, LEfSe and random forest to determine the biomarkers, the meaning of larval health indicators, and each health and stage condition. Biomarkers positively correlated with a given larval stage and health were selected and used to infer putative microbial functions in each condition. FAPROTAX (Functional Annotation of Prokaryotic Taxa) [50,51], a tool that predicts metabolic phenotypes and ecological features, as well as functions based on published data [50,52], was used via the microeco package [44]. To underline any role played by antibiotics or water treatment in larval microbiota, we performed an ANOSIM (analysis of similarity) using the vegan package [53] to indicate whether the microbial community of one group is statistically distinct from another group/other groups. ANOSIM results provide an R value close to 0 if all groups are close and cannot be separated, or close to 1 if dissimilarity between samples is observed. ANOSIM was performed using the vegan package.

## 3. Results

### 3.1. Main Genera Related to the Larval Stage and Health

**Figure 1:**
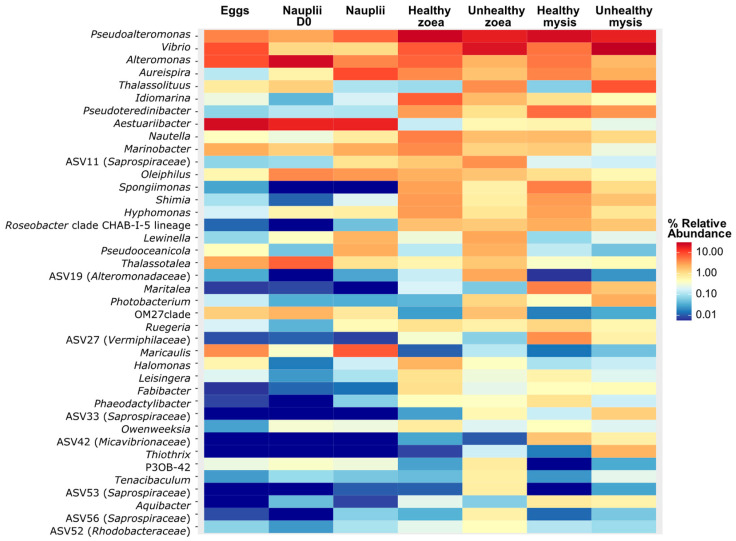
Heatmap of the top 40 genera spreading in the active larval microbiota according to larval stage and health.

Among the 40 main genera presented in Figure 1, *Aestuariibacter* and *Alteromonas* were predominant in both eggs and nauplii. Additionally, *Vibrio* and *Pseudoalteromonas* were abundant in the egg microbiota; *Thalassotalea* and *Oleiphilus* dominated in the nauplii prior to their transfer into the rearing tanks; and *Maricaulis* became prominent in the nauplii after one day in the tanks. *Pseudoalteromonas* and *Vibrio* were found to be abundant across both healthy and unhealthy zoea and mysis stages. *Alteromonas* and *Idiomaria* were also highly present in the microbiota of healthy zoea, while *Thalassolituus* was among the main genera in the unhealthy larvae. Finally, *Pseudoteredinibacter*, *Spongiimonas*, and *Maritalea* were abundant in the microbiota of healthy mysis. ANOSIM indicated that the water treatment (T or NT) did not influence the larval microbiota (R = 0.087, with a *p*-value at 0.0055); while the use of an antibiotic only slightly influenced the larval microbiota (R = 0.43, *p* = 0.0001).

### 3.2. Investigation of Biomarkers Related to the Larval Stage and Health as Indicators of Larval Health

**Figure 2:**
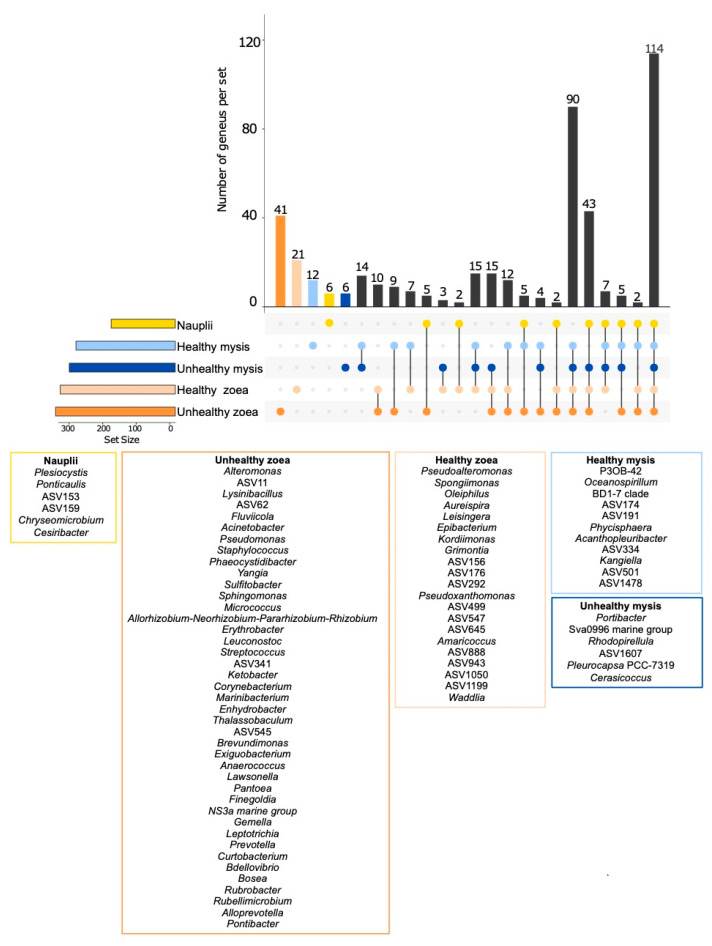
Specific genera enriched according to the larval stage and health based on the ANCOM-BC method. The Upset graph indicates bacterial genera that were unique or common to each group, namely, nauplii, healthy zoea, unhealthy zoea, healthy mysis, and unhealthy mysis, and the dots refer to the number of genera shared in one or several conditions. The top bar plot corresponds to the number of unique or shared genera in one or several groups, and it refers to the intersection of the Upset plot. The set size (on the left) corresponds to the number of genera in each group. Below are indicated the genera that are unique to each condition. Yellow refers to the nauplii, light blue to the healthy zoea, dark blue to the unhealthy zoea, light orange to the healthy mysis, and dark orange to the unhealthy mysis.

Biomarkers, as indicators of larval health, were investigated to identify bacterial genera associated with larval health status and to highlight those potentially involved in larval dysbiosis. Three methods were used—ANCOM-BC, LEfSe, and random forest—to compare healthy versus unhealthy zoea, as the mortalities started to occur at this stage, and to compare healthy and unhealthy mysis. ANCOM-BC, based on counts, displayed specific biomarkers for each condition (Figure 2). As displayed in Figure 2, a total of 41 genera were specific to the unhealthy zoea, 21 to the healthy zoea, 11 to the healthy mysis, and 6 to the unhealthy mysis. Meanwhile, 90 genera were common to both healthy and unhealthy zoea and mysis; and 114 genera were common across all conditions and were therefore present during all larval developmental stages, regardless of health status. Healthy zoea and healthy mysis shared seven common genera, whereas unhealthy zoea and mysis had no common genus.

**Figure 3:**
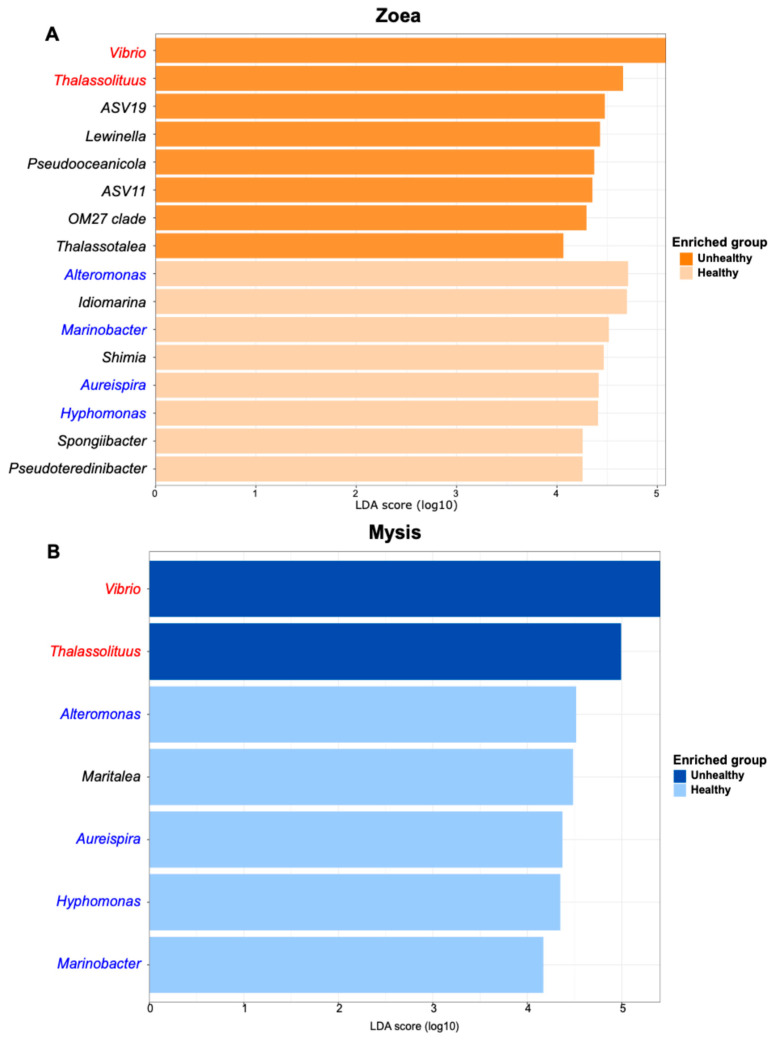
Biomarkers of the health status of the zoea and mysis stages detected by LEfSe, with an LDA threshold set at 4. (**A**) LEfSe displaying genera that were statistically more abundant in unhealthy zoea (in dark orange) and in healthy zoea (in light orange). (**B**) LEfSe displaying genera that were statistically more abundant in unhealthy mysis (in dark blue) and in healthy mysis (in light blue). Taxa in red were found in both unhealthy zoea and unhealth mysis; taxa in blue were found in both healthy zoea and mysis. The main LDA score indicates the statistical significance of genus enrichment in a given condition.

LEfSe analysis, based on statistical significance, displayed genera that were statistically more abundant according to the zoea or mysis stage and health status at a threshold set at four (Figure 3A,B). For zoea, LEfSe highlighted eight biomarkers of healthy zoea (*Alteromonas*, *Idiomarina*, *Marinobacter*, *Shimia*, *Aureispira*, *Hyphomonas*, *Spongiibacter* and *Pseudoteredinibacter*) and eight of unhealthy individuals with *Vibrio*, *Thalassolituus*, ASV19, *Lewinella*, *Pseudooceanicola*, ASV11, OM27 clade, and *Thalassotalea* (Figure 3A). *Alteromonas* and *Idiomarina* were the most statically enriched genera in the healthy zoea, while *Vibrio* and *Thalassolituus* were the most statically enriched genera in the unhealthy zoea. Considering the mysis stage, five bioindicators were related to the healthy mysis: *Alteromonas*, *Maritalea*, *Aureispira*, *Hyphomomas*, and *Marinobacter*, with *Alteromonas* and *Maritalea* being the most statistically enriched in this condition. Except for *Maritalea*, all were also biomarkers of the healthy zoea (Figure 3A,B). Only two biomarkers were related to the unhealthy mysis: *Vibrio* and *Thalassolituus* and were also detected as bioindicators of the unhealthy zoea.

**Figure 4:**
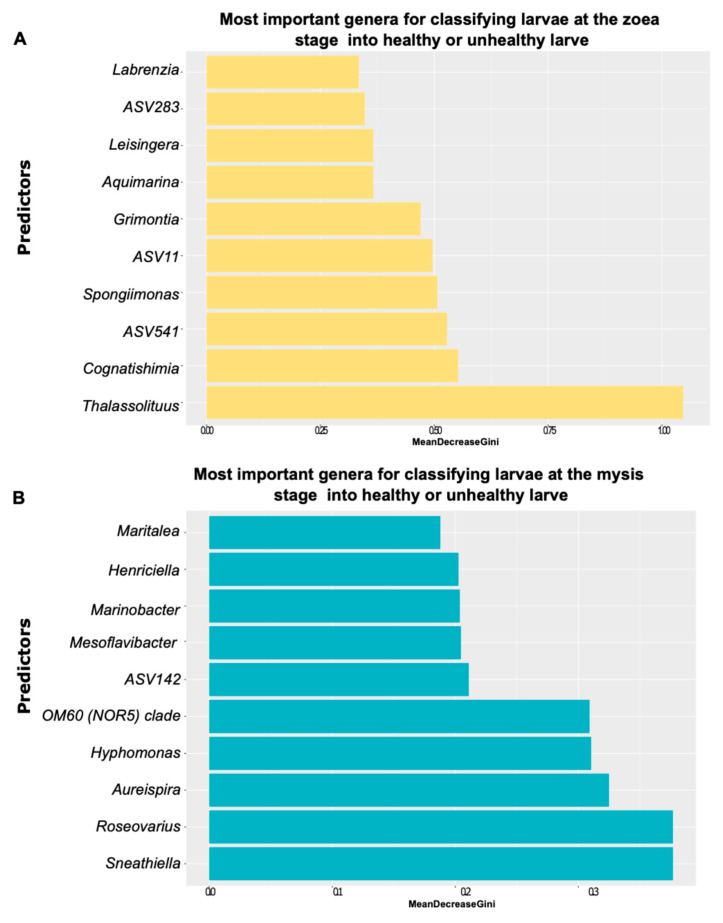
Biomarkers of the health status of the zoea and mysis stages detected via the random forest method. (**A**) Random forest displaying the 10 most important predictors at the genus level for distinguishing healthy from unhealthy zoea. (**B**) Random forest displaying the 10 most important predictors at the genera level for distinguishing healthy from unhealthy mysis. The Mean Decrease Gini or Gini-Based Variable Importance score indicates how important the predictor is for the model.

The random forest evidenced bacterial genera that were predictors of the larval survival for both zoea and mysis stages (Figure 4A,B). The main predictor (higher Mean Decrease Gini score) for distinguishing healthy zoea from unhealthy zoea was *Thalassolituus*, followed by *Cognathishimia* and the genera related to ASV52 (Figure 4A). Among the top ten predictors involved in the zoea classification into healthy or unhealthy larvae, two biomarkers, *Leisingera* and *Spongiimonas,* were also detected, with ANCOM-BC serving as a proxy for the zoea health. Additionally, ASV11 was confirmed by ANCOM-BC to be a biomarker of unhealthy zoea, and *Thalassolituus* was identified by LEfSe to be a marker of the unhealthy zoea and mysis. Among the predictors underlined to classify mysis into healthy or unhealthy larvae, *Sneathiella* and *Roseovarius* were the most important, followed by *Aureispira* (Figure 4B). Also, for mysis health prediction, three of the top ten predictors identified by random forest—*Marinobacter*, *Hyphomonas* and *Aureispira*—were also detected by LEfSe as biomarkers of healthy zoea and mysis. Finally, *Maritalea* was identified by LEfSe as a biomarker of healthy mysis, and it was among the important predictors highlighted by random forest (Figure 4A,B).

All biomarkers detected by the three methods were then evaluated for their correlation with a given health and stage using Spearman correlation analysis. Only three biomarkers were strongly positively (*p* ≤ 0.001) correlated with healthy zoea, namely, *Marinobacter*, *Idiomarina*, and *Mesoflavibacter*, while twenty-one bioindicators were strongly positively correlated (*p* ≤ 0.001) with the unhealthy zoea (Figure 4).

**Figure 5:**
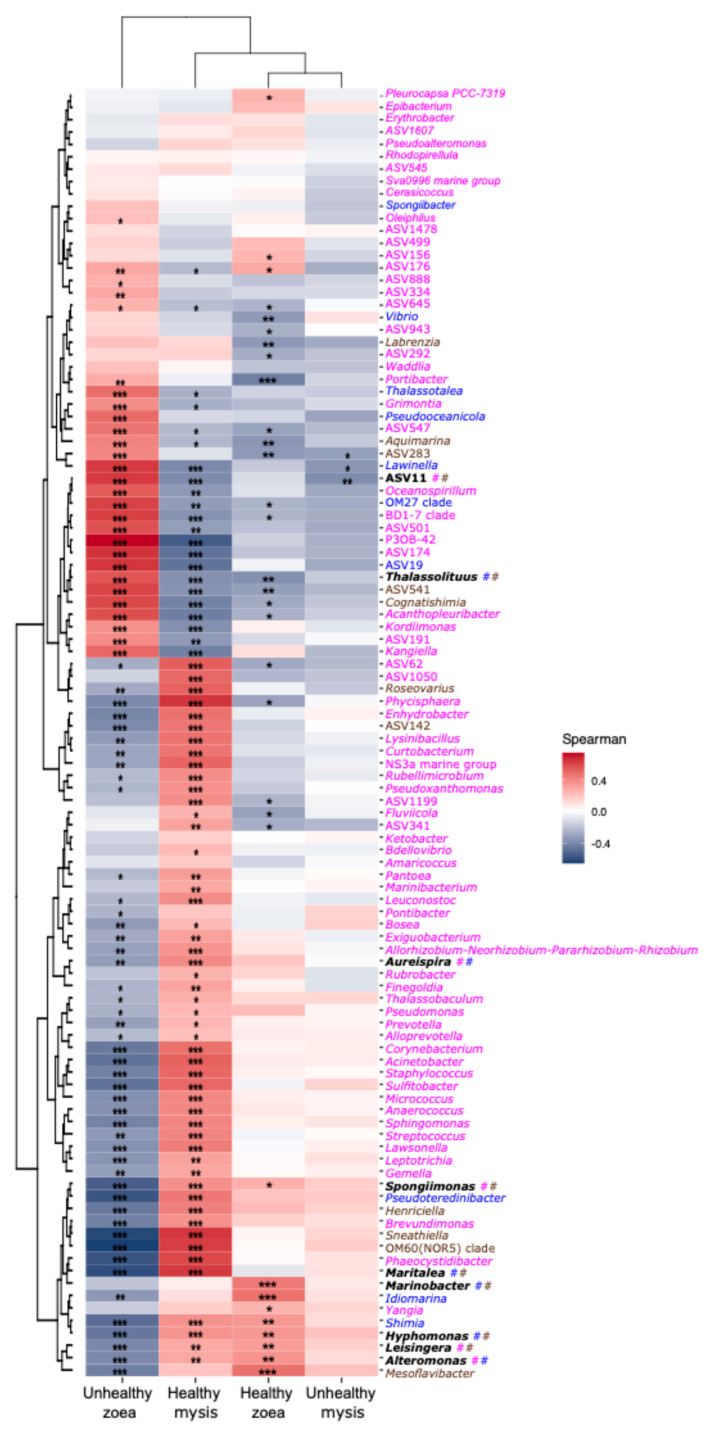
Correlogram of the biomarkers detected with ANCOM-BC, LEfSe, and random forest according to the larval stages and health status. The heatmap color gradient is linked to Spearman correlation coefficient intensity: red stands for positive correlation; while blue corresponds to negative correlation. Taxa in purple or followed by # were identified with the ANCOM-BC; taxa in blue or followed by # were identified with the LEfSe; taxa in brown or followed by # were identified with random forest; taxa in black (bold) were identified with two or three methods and the colored # indicated from which methods; . Non-significant correlations (*p* > 0.05) are noted with no asterisk, while significant correlations are represented with asterisks: *: *p* ≤ 0.05, **: *p* ≤ 0.01, ***: *p* ≤ 0.001.

The correlogram displayed 31 biomarkers strongly positively (*p* ≤ 0.001) correlated with healthy mysis (Figure 5). On the contrary, no real biomarkers for unhealthy mysis were in evidence as only weak positive correlations were detected (Figure 5).

### 3.3. Biomarkers and Putative Functions Associated with Larval Stage and Health

**Figure 6:**
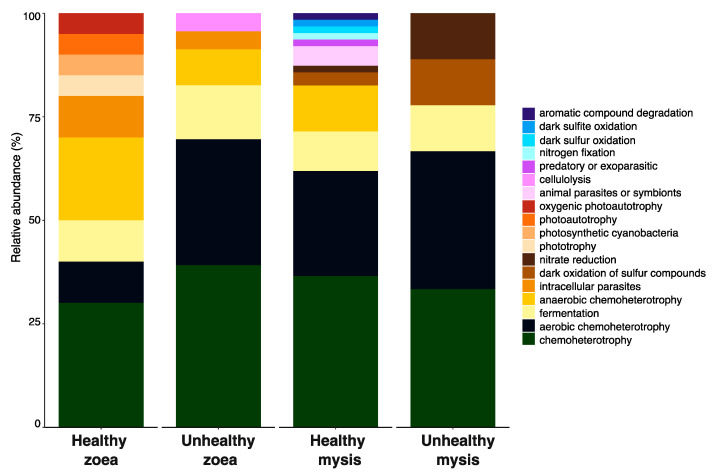
Relative abundance of the main putative ecological functions of the biomarkers positively correlated with larval stage and health status assigned via FAPROTAX.

Positively correlated biomarkers, as larval health indicators, for a given stage and health condition were then chosen to determine the putative enriched functions in each condition with FAPROTAX. Healthy zoea and healthy mysis conditions showed higher diversity of putative functions than the microbiota of unhealthy larvae (see Table S1 for the list of the detected bioindicators and their related putative function determined by FAPROTAX). Common putative functions, such as chemoheterotrophy, aerobic chemoheterotrophy, or fermentation, were found across all larval stages and health statuses (Figure 6). Different potential functions were also associated with specific biomarkers of a given condition (Figure 6). Putative functions such as phototrophy, photoautotrophy, and oxygenic photautotrophy were solely related to the biomarkers of the healthy zoea. Potential cellulolytic activity was detected only in the function related to the biomarkers of unhealthy zoea. Bacterial biomarkers of healthy mysis harbored putative functions related to animal parasites or symbionts, either predatory or exoparasitic; nitrogen fixation; dark sulfur and sulfite oxidation; and aromatic compound degradation. Biomarkers of the unhealthy mysis exhibited two potential functions, also found in healthy mysis but in lower abundance, namely, the dark oxidation of sulfur compounds and nitrate reduction (Figure 6). 

## 4. Discussion

Comparison of the active microbiota of healthy and unhealthy larvae at a given stage highlighted clear differences, indicating a probable dysbiosis of the microbiota of unhealthy individuals. Dysbiosis is characterized by a shift in microbial diversity, distribution, or metabolic activities, or a disruption of the microbiota, with a loss of beneficial or commensal taxa [13,14]. Consequently, genera associated with host health status can be evidenced through biomarker detection. To identify biomarkers involved in larval health regardless of the rearing method, we combined three methods: one based on count (ANCOM-BC, Figure 2), a second based on statistics (LEfSe, Figure 3A,B), and a third based on machine learning (random forest, Figure 4A,B). A comprehensive research approach was adopted to identify a broad range of biomarkers and to avoid missing any. Then, all enriched genera were correlated with a given larval stage and health status through Spearman correlations to untangle specific biomarkers as indicators of larval health, which could later be used as reliable proxies by which to monitor *Penaeus stylirostris* larvae in hatcheries, regardless of the rearing protocol. In this study, a unique protocol was used for all the samples, from RNA extraction and reverse transcription to sequencing and bioinformatic analysis, in order to minimize bias and enable sample comparison. An average of 100 larvae is typically used in shrimp larvae studies, enabling comparisons between studies [22,30,32,37,38,54,55]. The V4 hypervariable region of the 16S rRNA gene was chosen for multiple reasons. First, it is widely used in metabarcoding studies, including those related to shrimp or shrimp larvae [7,22,30,32,37,38,55]. Second, it is a rather stable prokaryotic 250pb region. According to García-López et al. (2020), using the V4 region enables the monitoring of prokaryotic dynamics and evolution and the development of tools for microbial biosurveillance [56]. In addition, care must be taken regarding the putative functions of biomarkers; the potential functions detected must be validated through further analysis, such as metatranscriptomics.

*Idiomarina*, *Mesoflavibacter*, and *Marinobacter* were the bioindicators associated with healthy zoea (Figure 4), meaning that they may play a beneficial role for the larvae. Members of *Idiomarina* are marine bacteria known to use amino acids and peptides as substrates [57]. They have been identified as biomarkers of healthy *P. stylirostris* larvae, where they might be involved in food acquisition [30]. *Mesoflavibacter* have been found to be beneficial bacteria of the intestine of healthy *Penaeus vannamei* juveniles, where they could be involved in toxin degradation or in zeaxanthin synthesis [58]. Zeaxanthin is a pigment known to have great pharmaceutical properties, such as antioxidant and human anti-cancer properties [59,60]. This genus has also been abundantly detected in the eggs and nauplii of *Penaeus indicus*, as well as in the gut of *Penaeus monodon* after *Vibrio harveyi* exposure [58,61]. Bacteria affiliated with *Marinobacter* are often associated with *P. stylirostris*, either as part of the core microbiota of healthy larvae or as the main genus constituting the egg and nauplii microbiota [30,37]. *Marinobacter* were identified as water biomarkers of healthy larvae, where they might exhibit a probiotic activity or, at least, an activity beneficial for the larvae [31]. In addition, *P. indicus* larvae fed with a microbial cocktail made of microalgae and bacteria affiliated with *Alteromonas*, *Labrenzia*, and *Marinobacter* had a better survival and growth rate [62].

Biomarkers of unhealthy zoea seemed to be divided into three groups, with some genera associated with diseased shrimp larvae, some with related healthy shrimps, and some able to prey on a broad spectrum of bacteria. Indeed, bioindicators affiliated with *Lewinella*, *Oceanospirillum*, and *Aquimarina* genera have often been detected in diseased shrimps. This could suggest a putative pathogenic or opportunistic role for these taxa [63,64,65]. In *P. vannamei* larvae affected by acute hepatopancreatic necrosis disease (AHPND), *Lewinella* was among the main genera detected in infected larvae [65]. In *P. vannamei* adults affected by *Vibrio parahaemolyticus* (strain that does not induce AHPND [64,66])*,* both *Lewinella* and *Oceanospirillum* were enriched in the gut microbiota of diseased shrimps [64]. Regarding the biomarker *Aquimarina*, species of this genus have been isolated from diseased *P. vannamei* shrimps, such as *Aquimarina hainanensis*; isolated from diseased larvae, or *Aquimarina penaei*; and isolated from the gut contents of diseased *P. vannamei* adults. Interestingly in crustaceans affected by shell disease, members of *Aquamarina* genus are often found in the epibacterial communities of affected carapace [63,67]. Contrary to these biomarkers, which were proxies of diseased or unhealthy larvae, others, such as *Cognatishimia*, *Thalassolituus*, and *Grimontia*, have been frequently detected in healthy *P. vannamei*, *P. stylirostris*, or *P. monodon* shrimp larvae [37,58,67,68]. In the post-larvae rearing of *P. vannamei* infected with AHPND, *Cognatishimia* were enriched in the microbiota of post-larvae collected in rearing tanks with high survival rates [54]. *Thalassolituus* have been found in the egg and nauplius microbiotas of *P. stylirostris* [37], as well as in the nauplius and zoea microbiota of *P. vannamei* [67]. Some *Thalassolituus* species are hydrocarbonoclasts [69] and might therefore be involved in food acquisition for the larvae. *Grimontia* have been detected in the microbiota of *P. monodon* as one of the stage-specific genera of mysis [70]. The BD1-7 clade was also a bioindicator of unhealthy zoea. *Kordiimonas* was also a biomarker of unhealthy zoea in this study, although its role is unclear as this genus was dominant in the *P. indicus* microbiota at the mysis stage [61], while it was a biomarker of unhealthy *P. vannamei* in both larvae and rearing water [22]. We can hypothesize that this observed discrepancy is related to *Kordiimonas* being species-specific or related to interactions with other bacterial lineages of the larval microbiota. P3OB-42 affiliated with the *Myxococcales* are predatory bacteria that can kill a broad range of microorganisms through a so called “wolfpack” predation [71]. We can hypothesize that the enrichment of P3OB-42 in the unhealthy zoea microbiota plays a role in the regulation of non-beneficial bacteria and in holobiont defense, especially against dysbiosis. The bioindicators of the unhealthy zoea revealed ongoing competition in their microbiotas. This gave us insight into the different steps that could occur before or during dysbiosis establishment. Indeed, enrichment of beneficial genera such as *Cognitishimia*, *Thalassolituus*, and *Grimontia* could help larval development, nutrition, and health. P3OB-42, with its predation feature, could act as a biocontrol against opportunistic or etiological agents; whereas *Lewinella*, *Oceanospirillum*, and *Aquimarina* seemed to act as non-beneficial, opportunist, or even putative pathogenic taxa that impaired larval health. This suggests that the zoea stage, where mortalities first occurred, is a key stage for shifts in microbial diversity and activity that can further lead to dysbiosis and therefore to a pathobiome [11,35]. Indeed, it is at this stage that great changes occurred for the larvae, notably mouth opening [72] accompanied by host-driven selection pressure. Further analysis, such as metatranscriptomics, should be conducted to decipher the microbiota structure and shift between healthy and unhealthy zoea, considering the three zoea sub-stages.

Biomarkers of the healthy mysis showed contrasting roles, with some being beneficial for the host and others being potential pathogens or opportunists. Among the beneficial taxa, we can cite *Phycisphaera*, *Maritalea*, *Roseovarius*, NS3a marine group, *Sulfitobacter*, *Lysinibacillus*, and *Curtobacterium*. *Phycisphaera* were found in the microbiota of healthy *P. monodon* nauplii, zoea, and mysis [71]. *Maritalea* were detected as bioindicators of healthy *P. stylirostris* mysis [30]. Members of the NS3a marine group were biomarkers in the rearing water of healthy *P. vannamei* larvae [22]. This suggests that members of the *Phycispharea*, *Maritalea*, and NS3a marine groups might support or improve animal health. *Sulfitobacter* was enriched in the gut of healthy *P. vannamei* shrimps, and its abundance decreased in diseased and moribund shrimps and seemed to protect the shrimps against *V. parahaemolyticus* infection [26]. Members of the *Sulfitobacter* genus also constituted the core microbiota of healthy *P. stylirostris* larvae, where they might act as probiotic or beneficial bacteria towards the host [30]. Some biomarkers seemed to have probiotic properties, such as *Lysinibacillus*. In shrimp fed with rice bran fermented with *Bacillus* and *Lysinibacillus*, the putative pathogenic taxa in the gut of *P. vannamei* were reduced and the growth and survival rates of the shrimps were improved [73]. *Lysinibacillus* was also used as a probiotic in *P. indicus* rearing hatcheries [74]. In addition, *Lysinibacillus fusiformis* can degrade chitin via chitinolytic activity [75], as well as some *Curtobacterium* species [76,77]. Used as a probiotic in the rearing water of juvenile *P. vannamei*, *Curtobacterium* sp. strain S13 increased the superoxide dismutase (SOD) activity in the muscle and therefore played a role in shrimp immunity [78]. *Pseudoteredinibacter* also seemed to be beneficial for the healthy mysis as this genus was previously identified as a biomarker of healthy *P. stylirostris* mysis [30]. *Roseovarius* could also be considered as a beneficial biomarker as it was positively correlated with healthy zoea. However, at the time of writing, there is no evidence of the putative beneficial or probiotic role of *Roseovarius* in shrimps. In contrast, several bioindicators appeared to be unbeneficial for the larvae such as *Sneathiella*, *Acinetobacter*, and *Lewsonella*. Indeed, *Sneathiella* was enriched in *P. vannamei* larvae affected by AHPND [54,65]. *Acinetobacter* was evidenced as a putative pathogen of the red leg disease of *P. vannamei* [79].

Regarding the mysis, *Phaeocystidibacter*, *Staphylococcus*, *Enhydrobacter*, *Corynebacterium*, and *Pseudoteredinibacter* appeared to have contrasting roles in mysis health; they could be beneficial or opportunist taxa. Indeed, *Phaeocystidibacter* was a biomarker in the rearing water of unhealthy *P. stylirostris* zoea but became a biomarker of healthy *P. stylirostris* mysis larvae in the rearing water [31]. *Staphylococcus* was among the main genera in the gut microbiota of *P. vannamei* when *Bacilli* and lactic acid bacteria were added in the rearing water, allowing for a better survival rate compared to the control [80]; whereas, in *P. vannamei* infected with AHPND, *Staphylococcus* was enriched in their gut microbiota [25]. The same trend was observed for *Corynebacterium,* which was enriched in the gut microbiota of AHPND-affected *P. vannamei* [25], while this genus was among the main genera in the gut microbiota of *P. vannamei* fed with honey prebiotic [28]. *Enhydrobacter* was found to be enriched in the gut of *P. vannamei* fed with food supplemented in 5-aminolevulinic acid, known to improve shrimp immune system [81]. *Enhydrobacter* was also abundant in *P. vannamei* affected with white feces syndrome [24]; while it decreased in *P. vannamei* gut when shrimps were exposed to glyphosate [82]. Such a discrepancy in the putative roles of the detected bioindicators underlines that the apparently healthy mysis could be struggling against abiotic or biotic factors, such as unappropriated conditions and opportunistic or ethologic agents. This could also indicate a potentially fatal fate for the mysis and therefore for the rearing. Also, the putative role of the necrobiome should be considered as it might influence both the rearing conditions and the microbiota associated with the rearing water and the larvae (alive and dead) [31,83,84].

No strong correlations between biomarkers and unhealthy mysis were found, suggesting that dysbiosis had affected the whole microbiota with no specific enriched genera (Figure 5). This could mean that the holobiont, as well as the host–microbiota interactions, were impaired. In addition, some unhealthy mysis did not come from unhealthy zoea, meaning that they became unhealthy later in the rearing process. This could indicate a second wave of detrimental factors (stress, pathogen, other), impacting larval health and explaining the lack of biomarkers for the unhealthy mysis. To better understand this process and the putative two-wave dysbiosis, i.e., one associated with zoea and the other to mysis, further work is required, such as characterizing the active microbiota across each zoea and mysis stage, along with meta-transcriptomic analysis.

To determine whether the listed biomarkers should be considered beneficial, detrimental, context-dependent (i.e., beneficial or harmful according to rearing health), or capable of preying on cells, further studies are required to clarify their actual roles in larval health and their ecological function during rearing. Metatranscriptomics analyses could help to resolve their functions during rearing. In addition, bacterial isolation and species description, along with genome sequencing and annotation, will help in the assessment of their metabolic activities, including their ability to produce secondary metabolites, to exhibit antimicrobial activities, or to prey on cells.

Using FAPROTAX, looking at the putative microbial activities of all the bioindicators positively correlated with a given larval stage and health, four functions were found in all healthy stages in both zoea and mysis: chemoheterotrophy, aerobic chemoheterotrophy, fermentation, and anaerobic chemoheterotrophy (Figure 6 and Table S1). Thus, that these functions were performed by various biomarkers seems important to the holobiont. In the same way, intracellular parasitic functions were shared among healthy and unhealthy zoea, while the dark oxidation of sulfur compounds and nitrate reduction were co-owned by the biomarkers of the mysis, regardless of their health status, exhibiting stage-specific functions, as we have previously shown [30]. Every health stage harbored its own functions except for the unhealthy mysis, which was probably due to the lack of positively correlated specific bioindicators. These data, along with the indicated biomarkers and their associated functions, indicate fewer host–microbiota interactions occurring in the unhealthy animals, suggesting that microbial dysbiosis affected the whole microbiota and its host. However, as FAPROTAX only provides potential functionality of the detected bioindicators, metatranscriptomic approaches could be considered to gain insight into the metabolic activities of the active microbiota and how they may interact with the host.

## 5. Conclusions

Overall, using a combination of three methods coupled with Spearman’s correlations, we were able to identify biomarkers that were strongly related to healthy and unhealthy zoea (three for the healthy and six for the unhealthy) as well as healthy mysis (eight bio-indicators). However, we were unable to identify biomarkers for unhealthy mysis. Our findings showed that the bioindicators associated with healthy zoea, namely, *Idiomarina*, *Mesoflavibacter*, or *Marinoabcter*, were potentially beneficial for the larvae. Biomarkers related to the unhealthy zoea were found to be associated with diseased shrimps, suggesting that the larvae might be struggling against detrimental abiotic and/or biotic conditions (opportunistic microorganisms, pathogens). The same observation was made for the unhealthy mysis. However, further studies are needed, such as metatranscriptomics, multi-omic approaches, or strains isolations and description (physiology, metabolism, and genome annotation), to prove that larval microbiota is indeed struggling against something harmful or against detrimental microorganisms. Such analyses will also reveal the transcripts, proteins, or enzymes involved in larvae homeostasis. Our findings also suggest that a two-wave dysbiosis process could occur during the rearing, one affecting the zoea and another affecting the mysis. Further work, based on omic-approaches, needs to be realized at each zoea and mysis sub-stage to better understand how the larval microbiotas are affected. This will allow us to highlight, on a finer scale, which microbial communities are involved, as well as their ecological functions. This should also underline the processes involved, such as selection pressure, role of the necrobiome, deterministic or stochastic mechanisms, etc. Furthermore, we identified biomarkers that might harbor probiotic activities in both healthy and unhealthy larvae. Isolating these strains and screening them for antimicrobial activities and secondary metabolites production could be useful for future research. Indeed, working on how to incorporate probiotics into larval food or rearing water could help to improve survival and to maintain both the larvae and the homeostasis of their microbiota. Finally, we revealed that host–microbiota interactions appeared to be influenced by health status and larval ontogeny. To conclude, despite the unknown ecological role of the detected biomarker, we managed to distinguish larval stage- and health-specific biomarkers. This is a first step in the development of a biosurveillance and early detection tool for preventing larval mortality in the hatcheries. Indeed, PCR and qPCR primers could be designed from the detected bioindicators and used to detect, quantify, and qualify them during large-scale rearings. Ultimately, the ratio between biomarkers related to healthy larvae and those detected in unhealthy larvae could be used to define thresholds and to help predict rearing outcomes.

## Data Availability

The raw 16S RNA data are available in Microlarve on the NCBI SRA repository under the Microlarve BioProject ID PRJNA736535 (BioSamples SAMN39924754 to SAMN39924780 include all the larvae samples reared with NT water and antibiotic added since D0 (NTA0 samples); BioSamples SAMN46319719 to SAMN66319742 encompass all the larvae samples reared with NT water without antibiotic (NTSA samples); BioSamples SAMN46321701 to SAMN66321823 gather all the larvae samples reared with T water without antibiotic (TSA samples); BioSamples SAMN46321806 to SAMN46321823 include all the larvae samples reared with NT water and antibiotic added since D3 (NTA3 samples); BioSamples SAMN46321788 to SAMN46321805 gather all the larvae samples reared with T water and antibiotic added since D3 (TA3 samples); samples M4_Egg1, M4_Egg2, M4_Nii1 (collected on D0), and M4_Nii2 (collected on D0) samples are available in SAMN19659075 and SAMN19659076, respectively).

## Supplementary Materials

The following supporting information can be downloaded at: https://www.mdpi.com/article/10.3390/microorganisms13112452/s1. Table S1: Metadata of the larval samples; Table S2: Detected bioindicators at the genus level, and their related putative function determined by FAPROTAX.
