# Supplementary material for: Potential Bacterial Biomarkers Associated with Penaeus stylirostris Shrimp Larvae to Infer Holobiont Health and Dysbiosis Across Larvae Stages
Source: Microorganisms. 2025 Oct 25;13(11):2452. doi: 10.3390/microorganisms13112452 (PMC12654795; doi:10.3390/microorganisms13112452)
Supplement: Supplementary file 1 [file microorganisms-13-02452-s001.zip › Table S1 pour mdpi.pdf]

S1 Table. Metadata of the larval samples.

Nii means nauplii collected on D0. A0 means that antibiotic (Erythromycin at 2 ppm) was first added on D0, and then on D3, D5, D7 and D9. A3 means that Erythromycin at 2 ppm was first added on D3, and then on D5, D7 and D9. SA means without antibiotics and L corresponds to larvae. Larval stage corresponds to the larval development observed during the morning.

NT means that the tanks were filled with non-treated water, which is seawater that that has gone through a sand filter and a 10 µm membrane filter before being stored in a reservoir with intensive bubbling (Callac et al., 2024). T means that the tanks were filled with treated water, which is seawater that has gone through a sand filter and a 10 µm membrane filter before being stored in a reservoir where the water was circulating through a filter and a series of filters (10 and 5 µm) during 3 days before being used to fill the rearing tanks (Callac et al., 2024).

Sample	Rearing day	Survival rate (%)	Larval stage	Antibiotics	Water type
**Egg1**	D-1	100	Egg	No	T
**Egg2**	D-1	100	Egg	No	T
**Nii1 D0**	D0	100	Nauplii	No	T
**Nii2 D0**	D0	100	Nauplii	No	T
**NTA0L_D1A**	D1	100,0	Nauplii	Yes on D0, D3, D5, D7 and D9	NT
**NTA0L_D1B**	D1	92,4	Nauplii	Yes on D0, D3, D5, D7 and D9	NT
**NTA0L_D1C**	D1	90,7	Nauplii	Yes on D0, D3, D5, D7 and D9	NT
**NTA0L_D2A**	D2	100	Zoea	Yes on D0, D3, D5, D7 and D9	NT
**NTA0L_D2B**	D2	89,8	Zoea	Yes on D0, D3, D5, D7 and D9	NT
**NTA0L_D2C**	D2	96,9	Zoea	Yes on D0, D3, D5, D7 and D9	NT
**NTA0L_D3A**	D3	96,3	Zoea	Yes on D0, D3, D5, D7 and D9	NT
**NTA0L_D3B**	D3	92,2	Zoea	Yes on D0, D3, D5, D7 and D9	NT
**NTA0L_D3C**	D3	98,0	Zoea	Yes on D0, D3, D5, D7 and D9	NT
**NTA0L_D4A**	D4	87,0	Zoea	Yes on D0, D3, D5, D7 and D9	NT
**NTA0L_D4B**	D4	96,7	Zoea	Yes on D0, D3, D5, D7 and D9	NT
**NTA0L_D4C**	D4	109,1	Zoea	Yes on D0, D3, D5, D7 and D9	NT
**NTA0L_D5A**	D5	82,6	Zoea	Yes on D0, D3, D5, D7 and D9	NT
**NTA0L_D5B**	D5	87,4	Zoea	Yes on D0, D3, D5, D7 and D9	NT
**NTA0L_D5C**	D5	91,1	Zoea	Yes on D0, D3, D5, D7 and D9	NT
**NTA0L_D6A**	D6	72,6	Zoea	Yes on D0, D3, D5, D7 and D9	NT
**NTA0L_D6B**	D6	73,3	Zoea	Yes on D0, D3, D5, D7 and D9	NT
**NTA0L_D6C**	D6	80,7	Zoea	Yes on D0, D3, D5, D7 and D9	NT
**NTA0L_D7A**	D7	78,1	Mysis	Yes on D0, D3, D5, D7 and D9	NT
**NTA0L_D7B**	D7	69,6	Mysis	Yes on D0, D3, D5, D7 and D9	NT
**NTA0L_D7C**	D7	66,5	Mysis	Yes on D0, D3, D5, D7 and D9	NT

**NTA0L_D8A**	D8	73,3	Mysis	Yes on D0, D3, D5, D7 and D9	NT
**NTA0L_D8B**	D8	72,8	Mysis	Yes on D0, D3, D5, D7 and D9	NT
**NTA0L_D8C**	D8	73,3	Mysis	Yes on D0, D3, D5, D7 and D9	NT
**NTA0L_D9A**	D9	75,9	Mysis	Yes on D0, D3, D5, D7 and D9	NT
**NTA0L_D9B**	D9	70,9	Mysis	Yes on D0, D3, D5, D7 and D9	NT
**NTA0L_D9C**	D9	66,5	Mysis	Yes on D0, D3, D5, D7 and D9	NT
**NTA3L_D4A**	D4	89,3	Zoea	Yes on D3, D5, D7 and D9	NT
**NTA3L_D4B**	D4	86,3	Zoea	Yes on D3, D5, D7 and D9	NT
**NTA3L_D4C**	D4	93,1	Zoea	Yes on D3, D5, D7 and D9	NT
**NTA3L_D5A**	D5	73,3	Zoea	Yes on D3, D5, D7 and D9	NT
**NTA3L_D5B**	D5	74,6	Zoea	Yes on D3, D5, D7 and D9	NT
**NTA3L_D5C**	D5	83,9	Zoea	Yes on D3, D5, D7 and D9	NT
**NTA3L_D6A**	D6	70,4	Zoea	Yes on D3, D5, D7 and D9	NT
**NTA3L_D6B**	D6	69,6	Zoea	Yes on D3, D5, D7 and D9	NT
**NTA3L_D6C**	D6	66,7	Zoea	Yes on D3, D5, D7 and D9	NT
**NTA3L_D7A**	D7	72,2	Mysis	Yes on D3, D5, D7 and D9	NT
**NTA3L_D7B**	D7	66,5	Mysis	Yes on D3, D5, D7 and D9	NT
**NTA3L_D7C**	D7	56,7	Mysis	Yes on D3, D5, D7 and D9	NT
**NTA3L_D8A**	D8	82,0	Mysis	Yes on D3, D5, D7 and D9	NT
**NTA3L_D8B**	D8	70,9	Mysis	Yes on D3, D5, D7 and D9	NT
**NTA3L_D8C**	D8	48,0	Mysis	Yes on D3, D5, D7 and D9	NT
**NTA3L_D9A**	D9	74,6	Mysis	Yes on D3, D5, D7 and D9	NT
**NTA3L_D9B**	D9	70,4	Mysis	Yes on D3, D5, D7 and D9	NT
**NTA3L_D9C**	D9	37,4	Mysis	Yes on D3, D5, D7 and D9	NT
**NTSAL_D1A**	D1	96,1	Nauplii	No	NT
**NTSAL_D1B**	D1	98,7	Nauplii	No	NT
**NTSAL_D1C**	D1	95,6	Nauplii	No	NT
**NTSAL_D2A**	D2	96,1	Zoea	No	NT
**NTSAL_D2B**	D2	96,9	Zoea	No	NT
**NTSAL_D2C**	D2	97,4	Zoea	No	NT
**NTSAL_D3A**	D3	86,9	Zoea	No	NT
**NTSAL_D3B**	D3	86,9	Zoea	No	NT
**NTSAL_D3C**	D3	93,7	Zoea	No	NT
**NTSAL_D4A**	D4	65,9	Zoea	No	NT
**NTSAL_D4B**	D4	69,6	Zoea	No	NT
**NTSAL_D4C**	D4	83,9	Zoea	No	NT
**NTSAL_D5A**	D5	40,0	Zoea	No	NT
**NTSAL_D5B**	D5	44,3	Zoea	No	NT
**NTSAL_D5C**	D5	59,8	Zoea	No	NT

**NTSAL_D6A**	D6	27,8	Zoea	No	NT
**NTSAL_D6B**	D6	22,8	Zoea	No	NT
**NTSAL_D6C**	D6	38,9	Zoea	No	NT
**NTSAL_D7A**	D7	17,2	Mysis	No	NT
**NTSAL_D7B**	D7	12,0	Mysis	No	NT
**NTSAL_D7C**	D7	21,5	Mysis	No	NT
**NTSAL_D8A**	D8	9,8	Mysis	No	NT
**NTSAL_D8B**	D8	5,9	Mysis	No	NT
**NTSAL_D8C**	D8	10,4	Mysis	No	NT
**TA3L_D4A**	D4	81,5	Zoea	Yes on D3, D5, D7 and D9	T
**TA3L_D4B**	D4	82,0	Zoea	Yes on D3, D5, D7 and D9	T
**TA3L_D4C**	D4	78,3	Zoea	Yes on D3, D5, D7 and D9	T
**TA3L_D5A**	D5	68,5	Zoea	Yes on D3, D5, D7 and D9	T
**TA3L_D5B**	D5	77,6	Zoea	Yes on D3, D5, D7 and D9	T
**TA3L_D5C**	D5	77,6	Zoea	Yes on D3, D5, D7 and D9	T
**TA3L_D6A**	D6	59,8	Zoea	Yes on D3, D5, D7 and D9	T
**TA3L_D6B**	D6	79,4	Zoea	Yes on D3, D5, D7 and D9	T
**TA3L_D6C**	D6	70,2	Zoea	Yes on D3, D5, D7 and D9	T
**TA3L_D7A**	D7	61,7	Mysis	Yes on D3, D5, D7 and D9	T
**TA3L_D7B**	D7	80,0	Mysis	Yes on D3, D5, D7 and D9	T
**TA3L_D7C**	D7	62,8	Mysis	Yes on D3, D5, D7 and D9	T
**TA3L_D8A**	D8	65,4	Mysis	Yes on D3, D5, D7 and D9	T
**TA3L_D8B**	D8	75,7	Mysis	Yes on D3, D5, D7 and D9	T
**TA3L_D8C**	D8	57,4	Mysis	Yes on D3, D5, D7 and D9	T
**TA3L_D9A**	D9	56,1	Mysis	Yes on D3, D5, D7 and D9	T
**TA3L_D9B**	D9	65,9	Mysis	Yes on D3, D5, D7 and D9	T
**TA3L_D9C**	D9	57,4	Mysis	Yes on D3, D5, D7 and D9	T
**TSAL_D1A**	D1	88,1	Nauplii	No	T
**TSAL_D1B**	D1	99,3	Nauplii	No	T
**TSAL_D1C**	D1	91,3	Nauplii	No	T
**TSAL_D2A**	D2	85,6	Zoea	No	T
**TSAL_D2B**	D2	101,7	Zoea	No	T
**TSAL_D2C**	D2	92,4	Zoea	No	T
**TSAL_D3A**	D3	80,0	Zoea	No	T
**TSAL_D3B**	D3	93,1	Zoea	No	T
**TSAL_D3C**	D3	91,1	Zoea	No	T
**TSAL_D4A**	D4	54,8	Zoea	No	T
**TSAL_D4B**	D4	83,9	Zoea	No	T
**TSAL_D4C**	D4	81,9	Zoea	No	T

**TSAL_D5A**	D5	27,0	Zoea	No	T
**TSAL_D5B**	D5	57,4	Zoea	No	T
**TSAL_D5C**	D5	53,5	Zoea	No	T
**TSAL_D6A**	D6	7,2	Zoea	No	T
**TSAL_D6B**	D6	41,3	Zoea	No	T
**TSAL_D6C**	D6	32,0	Zoea	No	T
**TSAL_D7A**	D7	3,5	Zoea	No	T
**TSAL_D7B**	D7	26,7	Zoea	No	T
**TSAL_D7C**	D7	18,5	Zoea	No	T
