# Supplementary material for: Potential Bacterial Biomarkers Associated with Penaeus stylirostris Shrimp Larvae to Infer Holobiont Health and Dysbiosis Across Larvae Stages
Source: Microorganisms. 2025 Oct 25;13(11):2452. doi: 10.3390/microorganisms13112452 (PMC12654795; doi:10.3390/microorganisms13112452)
Supplement: Supplementary file 1 [file microorganisms-13-02452-s001.zip › Table S2_Fraprotax genre et fonctions assocées.pdf]

Table S2: Detected bioindicators at the genus level, and their related putative function determined by FAPROTAX.

Genus	No function determined by FAPROTAX	Putative function(s) determined by FAPROTAX
Acanthopleuribacter		Aerobic chemoheterotrophy<br>Chemoheterotrophy
Acinetobacter		Aerobic chemoheterotrophy<br>Animal parasites or symbionts<br>aromatic compound degradation<br>chemoheterotrophy
Alloprevotella		Chemoheterotrophy<br>Anaerobic chemoheterotrophy
Allorhizobium-Neorhizobium-Pararhizobium-Rhizobium		Nitrogen fixation<br>Fermentation<br>aerobic chemoheterotrophy<br>chemoheterotrophy
Alteromonas		Fermentation<br>Chemoheterotrophy<br>aerobic chemoheterotrophy
Amaricoccus		Aerobic chemoheterotrophy<br>Chemoheterotrophy
Anaerococcus		
Aquimarina		Aerobic chemoheterotrophy<br>Chemoheterotrophy
**ASV1050 (*Rhodobacteraceae*)**	X	
**ASV11 (*Saprospiraceae*)**	X	
**ASV1199 (*Micrococcaceae*)**	X	
**ASV142 (*Saprospiraceae*)**	X	
**ASV1478 (NS9 marine group)**	X	
**ASV156 (UBA12409 family)**	X	
**ASV1607 (*Oxalobacteraceae*)**		Aerobic chemoheterotrophy<br>Chemoheterotrophy
**ASV174 (MBAE14 family)**		
**ASV176 (*Legionellaceae*)**		Intracellular parasites
**ASV19 (*Alteromonadaceae*)**		Fermentation<br>Chemoheterotrophy<br>anaerobic chemoheterotrophy
**ASV191 (*Rhodobacteraceae*)**	X	
**ASV283 (*Saprospiraceae*)**	X	
**ASV292 (*Alteromonadaceae*)**		Fermentation<br>Chemoheterotrophy<br>anaerobic chemoheterotrophy

**ASV334 (MBAE14 family)**	X	
**ASV341 (*Saprospiraceae*)**	X	
**ASV499 (*Fokiniaceae*)**		Intracellular parasites
**ASV501 (*Flammeovirgaceae*)**		Aerobic chemoheterotrophy<br>Chemoheterotrophy
**ASV541 (*Cryomorphaceae*)**	X	
**ASV545 (NS11-12 marine group)**	X	
**ASV547 (*Saccharospirillaceae*)**	X	
**ASV62 (*Terasakiellaceae*)**	X	
**ASV645 (*Saprospiraceae*)**	X	
**ASV888 (*Rhodobacteraceae*)**	X	
**ASV943 (*Rhodobacteraceae*)**	X	
Aureispira		Aerobic chemoheterotrophy<br>Chemoheterotrophy
**BD1-7 clade**		
Bdellovibrio		Predatory or exoparasitic
Bosea		Dark oxidation of sulfur compounds<br>Aerobic chemoheterotrophy<br>Chemoheterotrophy
Brevundimonas		Aerobic chemoheterotrophy<br>Chemoheterotroph
Cerasicoccus		Aerobic chemoheterotrophy<br>Chemoheterotroph
Cognatishimia	X	
Corynebacterium	X	
Enhydrobacter		Fermentation<br>Aerobic chemoheterotrophy<br>Animal parasites or symbionts<br>Chemoheterotrophy
Epibacterium		
Erythrobacter		Aerobic chemoheterotrophy<br>Chemoheterotrophy
Exiguobacterium	X	
Finegoldia	X	
Fluviicola	X	
Gemella		Fermentation<br>Chemoheterotrophy<br>Anaerobic chemoheterotrophy
Grimontia		Fermentation<br>Aerobic chemoheterotrophy<br>Chemoheterotrophy
Henriciella		Aerobic chemoheterotrophy<br>Chemoheterotrophy

Hyphomonas		Aerobic chemoheterotrophy<br>Chemoheterotrophy
Idiomarina		Fermentation<br>Chemoheterotrophy<br>Anaerobic chemoheterotrophy
Kangiella		Fermentation<br>Chemoheterotrophy<br>Anaerobic chemoheterotrophy
Ketobacter	X	
Kordiimonas	X	
Labrenzia		Aerobic chemoheterotrophy<br>Chemoheterotrophy
Lawsonella	X	
Leisingera	X	
Leptotrichia	X	
Leuconostoc		Fermentation<br>Chemoheterotrophy<br>Anaerobic chemoheterotrophy
Lewinella		Cellulolysis<br>Aerobic chemoheterotrophy<br>Chemoheterotrophy
Lysinibacillus	X	
Marinibacterium	X	
Marinobacter	X	
Maritalea		Aerobic chemoheterotrophy<br>Chemoheterotrophy
Mesoflavibacter		Chemoheterotrophy<br>Anaerobic chemoheterotrophy
Micrococcus		Aerobic chemoheterotrophy<br>Chemoheterotrophy
**NS3a marine group**		Chemoheterotrophy<br>Anaerobic chemoheterotrophy
Oceanospirillum		Aerobic chemoheterotrophy<br>Chemoheterotrophy
Oleiphilus	X	
**OM27 clade**		Predatory or exoparasitic
**OM6(NOR5) clade**	X	
**P3OB-42**	X	
Pantoea		Fermentation<br>Chemoheterotrophy<br>Anaerobic chemoheterotrophy
Phaeocystidibacter	X	
Phycisphaera	X	

***Pleurocapsa* PCC-7319**		Photosynthetic cyanobacteria<br>Oxygenic photoautotrophy<br>Photoautotrophy<br>Phototrophy
Pontibacter	X	
Prevotella		Animal parasites or symbionts
Pseudoalteromonas		Fermentation<br>Aerobic chemoheterotrophy<br>Chemoheterotrophy
Pseudomonas		Aerobic chemoheterotrophy<br>Chemoheterotrophy
Pseudooceanicola	X	
Pseudoteredinibacter	X	
Pseudoxanthomonas	X	
Rhodopirellula		Aerobic chemoheterotrophy<br>Chemoheterotrophy
Roseovarius		Aerobic chemoheterotrophy<br>Chemoheterotrophy
Rubellimicrobium	X	
Rubrobacter		Aerobic chemoheterotrophy<br>Nitrate reduction<br>Chemoheterotrophy
Shimia		Aerobic chemoheterotrophy<br>Chemoheterotrophy
Sneathiella	X	
Sphingomonas		Aerobic chemoheterotrophy<br>Chemoheterotrophy
Spongiibacter	X	
Spongiimonas		Chemoheterotrophy<br>Anaerobic chemoheterotrophy
Staphylococcus	X	
Streptococcus		Fermentation<br>Chemoheterotrophy<br>Anaerobic chemoheterotrophy
Sulfitobacter		Dark sulfite oxidation<br>Dark sulfur oxidation<br>Dark oxidation of sulfur compounds<br>Aerobic chemoheterotrophy<br>Chemoheterotrophy
**Sva996 marine group**	X	
Thalassobaculum	X	
Thalassolituus	X	

Thalassotalea		Chemoheterotrophy<br>Anaerobic chemoheterotrophy
Vibrio		Aerobic chemoheterotrophy<br>Nitrate reduction<br>Chemoheterotrophy
Waddlia		Intracellular parasites
Yangia	X	
